# To recruit or to graft? Comparing the recruitment of resident non-neuronal cells by lineage reprogramming with engraftment of stem cell-derived neurons for neuronal replacement therapies

**DOI:** 10.3389/fnins.2025.1589790

**Published:** 2025-05-21

**Authors:** Daniel A. Peterson

**Affiliations:** Center for Stem Cell and Regenerative Medicine and Center for Neurodegenerative Diseases and Therapeutics, Chicago Medical School, Rosalind Franklin University of Medicine and Science, North Chicago, IL, United States

**Keywords:** lineage reprogramming, stem cell therapeutics, cell grafting, gene therapy, neural repair and regeneration, CNS repair, induced pluripotent stem cell (iPSC), human embryonic stem cell (hESC)

## Abstract

Neurons are post-mitotic cells that are not replaced once lost, leading to the need for neuronal replacement therapies for central nervous system (CNS) repair. The generation of induced pluripotent stem cell (iPSC) derived human neurons is relatively advanced, with the capacity to generate pure and validated populations of different neuronal subtypes as clinical grade cells ready for engraftment. Clinical trials using human-derived embryonic stem cells (hESC) and iPSC-derived neurons are underway. As an alternative approach, the ability to target *in vivo* resident non-neuronal cells with reprogramming factors to induce replacement neurons has been demonstrated. The ability to engineer a defined population of resident replacement neurons that retain their cytoarchitectural location may permit an additional, more focused therapeutic strategy for specific circuits that could complement the bulk engraftment of ex vivo stem cell-derived replacement neurons. This mini-review summarizes and compares these two strategies and offers a perspective on the steps needed to advance recruitment as a complementary therapeutic strategy.

## Introduction

Most adult tissues contain some endogenous stem or progenitor cell populations that contribute to tissue maintenance and repair throughout life. However, some tissues, notably the neurons of the central nervous system, are already generated by birth and are long-lived cells. With limited exceptions, neurons are not replaced in the human brain or spinal cord, contributing to neurological deficits in the case of injury or disease. However, the adult human brain does maintain spatially restricted neurogenesis in the hippocampal dentate gyrus, and possibly the striatum, throughout life (Eriksson et al., 1998; Spalding et al., 2013). Although there have been reports to the contrary, possibly related to the extent of the post-mortem interval in samples (Terstege et al., 2022), the spatially restricted adult hippocampal neurogenesis is, nonetheless, unlikely to contribute new neurons to distant sites in need of repair (Bazarek and Peterson, 2014).

As a result, efforts have long been made to develop neuronal replacement therapies, beginning with neural grafting studies using chromaffin cells or fetal neurons (Niijima et al., 1995; Brundin et al., 1986; Gage and Björklund, 1986; Shetty and Turner, 2000). Delivery of human fetal neurons has had success, including improving symptoms in Parkinson’s patients (Mendez et al., 2008; Barker et al., 2013; Lia et al., 2016; Hallett et al., 2014), but is complicated by ethical considerations regarding the tissue source. The use of human embryonic stem cell (hESCs) as a cell source has also met with successs (Tabar et al., 2025; Li et al., 2024), but this approach also shares similar ethical considerations. Furthermore, both fetal and hESC-derived cells are allogeneic (same species), not autologous (same individual), and subject to concerns about safety and immunogenicity. Nonetheless, safety and immunogenicity concerns using allogeneic grafts may be successfully addressed (Emborg et al., 2025), particularly if donor cells are matched in regard to human leukocyte antigenicity (HLA) to the patient (Sawamoto et al., 2025; Yoshida et al., 2023). Another promising neuronal replacement approach is the *in vitro* generation of neural organoids. Organoids can be generated from both ESCs and iPSCs and, if left unguided, they will contain both neuronal and non-ectodermal cells (Lancaster et al., 2013). Alternatively, organoids can be developmentally guided to recreate a regional identity containing neurons with cell type-specific identities and other non-neuronal cells (Pasca et al., 2025; Revah et al., 2022; Velasco et al., 2019). Such approaches may be particularly useful in creating functional bridges and replacement circuits for spinal cord injury (Qiang et al., 2023).

An additional direction was provided by the breakthrough demonstrating that differentiated somatic cells could be turned into human induced pluripotent cells (iPSCs) (Takahashi et al., 2007). As iPSCs can be directed in their cell fate to differentiate into any tissue cell type, they offer enormous possibilities for regenerative therapies with the added value that the source cells are autologous, as they are generated from the patient’s own cells (Kim et al., 2022). Progress has been made with directed differentiation of iPSCs into various neuronal subtypes, including using forced expression of transcription factors for forward programming of cell lineage (Tsunemoto et al., 2015; Tsunemoto et al., 2018; Nakamura et al., 2023). In addition to their potential to reveal insights into neurological disease, the resulting new neurons could be grafted into the brain or spinal cord as replacement neurons. Indeed, clinical trials of iPSC-derived neuronal grafts for potential CNS repair have been conducted and are currently underway (Sawamoto et al., 2025; Parmar, 2018; Kirkeby et al., 2023; Takahashi, 2020), reviewed in Svendsen and Svendsen (2024). Progress has also been made in specifying iPSC-derived glial cells (Ehrlich et al., 2017), but this mini-review will focus on neuronal replacement strategies.

**Figure 1:**
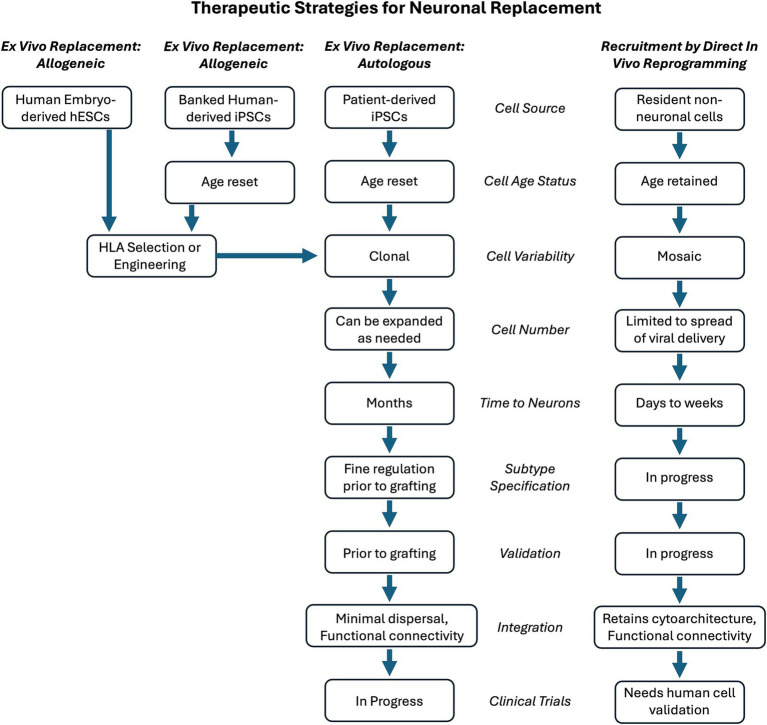
Comparison of engraftment or replacement strategies for neuronal replacement therapy. Therapeutic ex vivo cell engraftment could use cell sources that are allogeneic (same species) or autologous (same individual). To evade an immune response, allogeneic cells could be selected and HLA-matched for the recipient. This would facilitate the use of human-derived embryonic stem cells (hESCs) as one allogeneic cell source. Human induced pluripotent stem cells (iPSCs) could be used from a general bank of HLA-identified iPSCs that could serve as a universal donor when matched against the patient to receive them. Alternatively, a patient’s own iPSC cells could be generated, providing an autologous source for therapeutic engraftment. An additional therapeutic strategy for neuronal replacement is to recruit resident non-neuronal cells by in vivo lineage reprogramming them to become neurons. These replacement neurons would be directly in the location and circuitry in need of repair. The choice of strategy results in many steps needed to achieve the desired outcome. The features of the steps are compared for engraftment with recruitment strategies. The notation of “in progress” reflects the fact that the engraftment of stem cell-derived replacement neurons is a more developed and validated strategy. The recruitment of resident non-neuronal cells as replacement neurons requires further investigation to determine if it will meet its promise as an approach for CNS repair.

Using a similar approach to induced pluripotency, it has also been demonstrated that somatic cells can be directly reprogrammed into neurons (Vierbuchen et al., 2010). This includes recruiting non-neuronal cells resident in the CNS by delivering reprogramming factors. While reprogramming cell lineage shares advantages with induced pluripotency followed by directed differentiation, *in vivo* reprogramming differs in that the recruited replacement neurons remain resident within the regional cytoarchitecture, such as neocortex (Heinrich et al., 2014; Puglisi et al., 2024; Gascon et al., 2016; Bazarek et al., 2023), entorhinal cortex (Bazarek et al., 2023), striatum (Niu et al., 2015; Niu et al., 2013; Torper et al., 2013), hippocampus (Klempin et al., 2012; Lentini et al., 2021), or spinal cord (Tai et al., 2021). As the targeted cells are resident in the CNS, the extent of their recruitment is largely governed by the distribution of the vector used to deliver the reprogramming factors. Thus, the newly induced neurons, while being a more discreet population rather than a bulk engraftment, continue their existence in the cytoarchitecture where they were previously resident. A therapeutic potential can be envisioned whereby specific brain circuitry could be augmented by replacement neurons sourced from resident non-neuronal cells. This mini-review summarizes the progress in direct neuronal reprogramming and offers a perspective on next directions and the therapeutic potential of this additional strategy compared to hESC- or iPSC-sourced cells. Figure 1 presents a summary and comparison of these two approaches.

### Goals for neuronal replacement

A high priority for cell or tissue replacement therapies is evading immunological surveillance of foreign cells, tissues, or vectors and the resulting inflammatory response. Such a response may endanger the recipient and be detrimental to grafted cell survival or function. Studies in non-human primates have addressed management of the immune response in both allogeneic and allograft models [reviewed in Emborg et al. (2025)]. Recent reports of hESC (Tabar et al., 2025) and allogeneic iPSC (Sawamoto et al., 2025) clinical trials have been successful, suggesting that safety concerns can be managed. However, any strategies that can utilize a patient’s own cells for repair will have a distinct advantage. Both exogenous grafting of autologous iPSC-derived cells and recruitment of resident, endogenous cells by direct lineage reprogramming meet this criterion for personalized medicine.

Given the diversity of neuronal function and circuitry in the human CNS, there may be an advantage to utilizing therapeutic strategies with different approaches for different circumstances. For example, the decline and/or loss of regulated dopamine release that is a central deficit in Parkinson’s disease, could be successfully addressed by introducing a bulk population of appropriate iPSC-derived dopaminergic neurons. Other neurological deficits may benefit from recruiting induced neurons that are spatially integrated into an existing structured circuit to restore circuit output, for example following a focal stroke, traumatic brain injury, or to reset inhibitory tone in a region with epileptic activity (Lentini et al., 2021).

Many of the neurological deficits for which neuronal replacement therapies are desired occur in older patients. Thus, another consideration for the survival and functional integration of replacement neurons is the milieu into which they are placed. Engraftment into neonatal CNS can lead to impressive integration and dispersion of grafted cells (Windrem et al., 2014; Sim et al., 2011; Windrem et al., 2008), while mature adult CNS and aging CNS do not support such exuberant dispersion. For all cell replacement therapies, the extent of age-related environmental changes and the presence of disease pathophysiology and/or injury will also likely be important factors for successful repair.

### Considerations for exogenous cell replacement

The many advances in our understanding of iPSC generation and the subsequent directed cell fate to the desired neuronal subtype have made the use of iPSC-derived neurons a primary focus for therapeutic development. While such cells retain the patient’s genotype, including expression of genes that may be relevant to the pathophysiology the therapy is intended to address, the process of inducing pluripotency to rewind the cell to an embryonic stem cell-like state may provide enough of a reset to minimize the vulnerability of the newly induced neuron (Mertens et al., 2016). Although such a reset may hinder progress in using iPSC-derived neurons for studying disease pathophysiology, it would confer an advantage for neuronal replacement therapy. This would be particularly true for conditions where accumulation of pathophysiology may take decades in human patients.

Another advantage of using iPSC-derived neurons for therapies is the clonality, control over neuronal subtype specification, and validation that is possible in preparing cells for therapeutic delivery. The initial selection of iPSC colonies creates a homogenous, clonal population that should contribute to reduced variability in the cells to be grafted. Directed differentiation procedures have been carefully standardized and could also be augmented by forward programming approaches to produce a highly specific neuronal subtype for engraftment (Tsunemoto et al., 2015). Finally, it is possible to validate the phenotype, function, and purity of the newly generated neurons and to prepare precise numbers for the engraftment procedure (Kirkeby et al., 2023; Hiller et al., 2022). Concerns about safety and immunogenicity of iPSC-derived cell engraftment, even when allogeneic, has been mitigated by successful patient outcomes of a Phase I/II clinical trial for Parkinson’s disease (Sawamoto et al., 2025; Yoshida et al., 2023).

However, this high degree of control over the process of converting a patient’s own cells to make neurons for therapeutic delivery back to that patient comes at a cost. Improvements in process and control and the eventual scaling up of cGMP production facilities will reduce production costs and increase availability. The use of HLA selected or engineered allogeneic cells that can evade the immune response and be banked as a “universal” donor cell may lower the cost of therapeutic delivery (Yoshida et al., 2023; Tsuneyoshi et al., 2024; Maiers et al., 2025). Nevertheless, this process of producing clinical grade cells is resource intense and will likely remain an expensive procedure (Kirkeby et al., 2023). This may effectively limit the number of patients who, at a global level, can take advantage of truly autologous iPSC-derived neuronal engraftment as a promising therapeutic option.

There is also the consideration that the neurosurgical engraftment procedure may be quite invasive, possibly requiring multiple sites of delivery depending on the therapeutic requirements. In addition, the cells will be delivered in bulk with tens of thousands and possibly up to hundreds of thousands of cells being deposited at multiple sites (Kirkeby et al., 2023; Hiller et al., 2022). This may well meet the need for some neurological conditions, such as stroke or spinal cord injury, where a suitable cavity or space to receive this bulk delivery may exist secondary to the injury or in cases where the therapeutic efficacy of new neurons may be achieved without the need to infiltrate the surrounding parenchyma and physically integrate into remaining circuitry. However, in conditions without such injury-induced space, the bulk engraftment of cells may create its own complication as the space occupied could negatively impinge on adjacent tissue or elevate intracranial pressure. The extent to which this could be a concern would vary between conditions. Therefore, it is worth considering if alternative approaches exist that may offer distinct advantages for certain conditions where the need for neuronal replacement may be better served by a more limited and spatially distributed population of new neurons that could be integrated into existing circuits.

### Recruiting endogenous cells for reprogramming

An additional neuronal replacement strategy has been investigated based upon the finding that lineage committed non-neuronal cells can be directly reprogrammed into neurons without the induction of pluripotency (Vierbuchen et al., 2010). This raises the possibility of targeting non-neuronal cells already resident in the CNS and forcing their lineage to be changed to a neuronal lineage. To achieve this, the initial goals are the ability to target the desired cell and the ability to change its lineage. Implicit in the second goal is the need to control the subtype specification of the new neuron. Once this can be reliably achieved, the next set of goals is to establish the structural and functional integration of the newly generated neuron into existing circuitry, ultimately requiring the demonstration of their contribution to overall CNS function. Finally, the duration of the newly induced neurons should be determined.

Achieving all of these goals would provide the basis for translational studies demonstrating recruitment of endogenous cells for neuronal replacement in humans. While this recruitment approach is less developed compared to the relatively mature strategy of replacement therapy with iPSC-derived neurons, a number of reports support the initial goals of direct neuronal reprogramming as a strategy for neuronal replacement. Using gene delivery of transcription factors and other elements, direct *in vivo* neuronal reprogramming has been reported for astrocytes (Puglisi et al., 2024; Marichal et al., 2024), oligodendrocyte progenitor cells (OPCs) (Heinrich et al., 2014; Bazarek et al., 2023; Tai et al., 2021; Pereira et al., 2017; Torper et al., 2015), and microglia (Irie et al., 2023), reviewed in (Torper and Gotz, 2017). Mature oligodendrocytes have not been targeted due to the rational that it would be counterproductive to take them away from their important CNS function of myelination.

The neural cell populations most targeted, astrocytes and OPCs, are not monolithic and have considerable heterogeneity as a result of developmental origin, regional identity, age, and disease state (Heo et al., 2025; Bocchi et al., 2025; Sadick et al., 2022; Vigano and Dimou, 2016). Thus, recruiting the different non-neuronal cell populations already resident in the CNS may result in newly induced neurons with subtle differences, a possibility that needs further investigation. Another consideration for choosing a cell type to target is that the impact of re-tasking cell identity away from its current role may have functional implications. This concern has been raised for the targeting of astrocytes, as astrocytes perform critical roles in the CNS milieu and cellular homeostasis (Verkhratsky and Nedergaard, 2018). Furthermore, astrocytes seldom replace themselves by dividing in the absence of an injury stimulation raising the possibility that their large-scale recruitment could deplete their number and compromise local function. The most frequently dividing population in the mature CNS are the OPCs, that maintain a routine turnover in their population and increase proliferation in response to injury. In addition to their potential to terminally differentiate into oligodendrocytes, OPCs are reported to facilitate neuronal function (Fang et al., 2025) and contribute to synaptic remodeling (Auguste et al., 2022). Thus, while the full role of OPC function is still being revealed, their steady proliferation suggests that OPCs would not be depleted as they will be readily replaced if recruited into new neurons. This suggests that OPCs may be the optimal neural population to target for neuronal reprogramming. Microglia represent another potential population to target, but less is currently known about their suitability for recruitment.

The differences between non-neuronal cell populations also have implications for their specific targeting. Gene delivery has been extensively used for direct *in vivo* reprogramming studies, with adeno-associated viral (AAV) vectors initially being widely used. Although there are relative tropism differences among the various AAV serotypes, this targeting is not absolute, requiring the additional use of cell lineage-specific promoters to limit expression of the reprogramming factors to the desired target cell type. However, in a cautionary tale for the importance of validation, it was found that use of AAV resulted in off-target expression in already existing neurons so conclusions about neuronal induction could not be upheld (Puglisi et al., 2024; Cooper and Berninger, 2022; Calzolari and Berninger, 2021; Wang et al., 2021; Chen et al., 2022). As a result, investigations have turned to other vectors. Given that retroviral vectors only infect dividing cells and that almost all dividing cells in the naïve adult brain are OPCs, retrovirus has been used to effectively deliver reprogramming genes to OPCs (Puglisi et al., 2024; Gascon et al., 2016; Bazarek et al., 2023; Lentini et al., 2021; Marichal et al., 2024). As astrocytes primary are dividing in the neonatal brain, retroviral delivery to target astrocytes is optimally studied in the neonatal brain (Marichal et al., 2024). For the mature brain, there is a requirement of pre-injury to induce subsequent astrocyte proliferation (Puglisi et al., 2024).

Neurons are diverse and effective repair may even require replacement of more than one subtype of neuron. *In vitro* generation of iPSC-derived neurons readily permits directed differentiation of different neuronal subtypes, which potentially could be combined in an anticipated ratio prior to engraftment to achieve functional repair. However, control of the distribution of the replacement neurons is lost after engraftment at the desired location. In contrast, gene delivery for direct *in vivo* reprogramming allows more control over the cytoarchitectural locations to engineer replacement neuron contribution to circuit repair. To date, the range of neuronal subtypes reported include glutamatergic (Gascon et al., 2016; Tai et al., 2021; Torper et al., 2015) and GABAergic (Lentini et al., 2021; Tai et al., 2021) with additional subtype specification as calretinin- or parvalibumin-positive induced neurons (Niu et al., 2013; Pereira et al., 2017; Marichal et al., 2024). The choice of reprogramming transcription factor appears to be a primary driver of subtype specification with Ngn2 leading to excitatory induced neurons (Gascon et al., 2016; Bazarek et al., 2023) and Ascl1 leading to inhibitory induced neurons (Niu et al., 2015; Lentini et al., 2021; Tai et al., 2021; Pereira et al., 2017; Marichal et al., 2024). However, different specification outcomes have been reported (Tai et al., 2021; Torper et al., 2015). Nevertheless, this recruitment strategy is currently limited in the ability to continue to direct neuronal subtype specification once the initial gene delivery is made. The development of new approaches to modify newly reprogrammed neurons and/or their environment are needed to achieve the needed neuronal subtype specification of recruited replacement neurons.

### Next steps for developing a recruitment strategy for neuronal replacement therapies

A therapeutic recruitment strategy offers the ability to more precisely control the location, connectivity, and extent of replacement neurons and could provide a wider range of therapeutic options than offered by engraftment of stem cell-derived neurons alone. While progress has been made on the first two goals for recruiting resident non-neuronal cells using a direct *in vivo* reprogramming strategy (targeting the desired cell and reprogramming it into a neuron), further refinements in efficiency and subtype specification are needed to move this strategy forward. Some reports suggest functional integration of directly reprogrammed neurons can be achieved (Heinrich et al., 2014; Niu et al., 2013; Lentini et al., 2021; Pereira et al., 2017; Torper et al., 2015; Marichal et al., 2024), but additional evidence of circuit integration and activity is needed.

Despite this initial progress, there remain several challenges to the approach of direct reprogramming that require further investigation. The efficiency of neuronal recruitment, both in terms of number of cells targeted and successful lineage conversion, is low (Gascon et al., 2016; Bazarek et al., 2023; Marichal et al., 2024), particularly in the context of neuronal replacement by engraftment of thousands of cells. While the argument can be made that certain therapies may require a more subtle integration rather than bulk delivery, the current state of efficiency must still be improved. Greater precision in targeting non-neuronal cells and enhanced delivery volume of vectors should be developed. Given the potential safety risk of integrating viral vectors, the use of non-viral vectors should be investigated. Improvement in lineage conversion will require further insight into the transcriptional response and exploration of additional genetic or epigenetic manipulations to reduce cell loss through unsuccessful reprogramming (Gascon et al., 2016). Epigenetic barriers should be identified and environmental enhancement, such as co-delivery of trophic factors may also be important for long-term survival and integration of newly induced neurons (Niu et al., 2013; Grande et al., 2013).

To advance relevance for translational studies, the impact of neuronal replacement in the context of aging and/or pathophysiology related to injury or disease needs to be further explored. In particular, the duration of the newly-induced neuron function in aging and disease conditions needs to be determined. This will be important for both engraftment and recruitment strategies. Finally, recruitment studies will need to shift from reprogramming non-neuronal cells in rodent models to reprogramming human non-neuronal cells (Torper et al., 2013). This presents a particular challenge that could be met by generating the target human non-neuronal cell population *in vitro*. Once authenticated, reprogramming factors could be delivered to the human cells after engraftment to a host brain to mimic a therapeutic approach. In this case, the host brain (rodent or non-human primate) would serve as a surrogate for the human brain and provide insight that could justify clinical trials for replacement strategies. Although the recruitment strategy for neuronal replacement is not as developed as grafting strategies, with continued effort, it may offer an important additional therapeutic option for CNS repair.
